# Long non-coding RNA *MIR22HG* promotes osteogenic differentiation of bone marrow mesenchymal stem cells via PTEN/ AKT pathway

**DOI:** 10.1038/s41419-020-02813-2

**Published:** 2020-07-30

**Authors:** Chanyuan Jin, Lingfei Jia, Zhihui Tang, Yunfei Zheng

**Affiliations:** 1 The Second Clinical Division of Peking University School and Hospital of Stomatology, 100081 Beijing, China; 2 Central Laboratory, Peking University School and Hospital of Stomatology, 100081 Beijing, China; 3 Department of Oral and Maxillofacial Surgery, Peking University School and Hospital of Stomatology, 100081 Beijing, China; 4 Department of Orthodontics, Peking University School and Hospital of Stomatology, 100081 Beijing, China

**Keywords:** Bone development, Stem-cell differentiation

## Abstract

Osteoporosis is a prevalent metabolic bone disease characterized by low bone mineral density and degenerative disorders of bone tissues. Previous studies showed the abnormal osteogenic differentiation of endogenous bone marrow mesenchymal stem cells (BMSCs) contributes to the development of osteoporosis. However, the underlying mechanisms by which BMSCs undergo osteogenic differentiation remain largely unexplored. Recently, long non-coding RNAs have been discovered to play important roles in regulating BMSC osteogenesis. In this study, we first showed *MIR22HG*, which has been demonstrated to be involved in the progression of several cancer types, played an important role in regulating BMSC osteogenesis. We found the expression of *MIR22HG* was significantly decreased in mouse BMSCs from the osteoporotic mice and it was upregulated during the osteogenic differentiation of human BMSCs. Overexpression of *MIR22HG* in human BMSCs enhanced osteogenic differentiation, whereas *MIR22HG* knockdown inhibited osteogenic differentiation both in vitro and in vivo. Mechanistically, *MIR22HG* promoted osteogenic differentiation by downregulating phosphatase and tensin homolog (PTEN) and therefore activating AKT signaling. Moreover, we found *MIR22HG* overexpression promoted osteoclastogenesis of RAW264.7 cells, which indicated that *MIR22HG* played a significant role in bone metabolism and could be a therapeutic target for osteoporosis and other bone-related diseases.

## Introduction

Osteoporosis is one of the most common metabolic bone diseases which results from the disrupted balance between bone formation and resorption^1^. With the progressive aging of the global population, the incidence of osteoporosis is increasing dramatically which leads to an increased risk of fractures and exerts a strong impact on morbidity, and even mortality^2,3^. However, the pathological mechanisms of osteoporosis have not been fully understood yet. BMSCs are precursors that can differentiate into osteoblasts and adipocytes in bone and play an important role in maintaining bone homeostasis. Previous studies showed the impaired osteogenic differentiation capacity of BMSCs contributes to the progression of osteoporosis^4^. However, the molecular basis involved in the osteogenic differentiation of BMSCs remains unclear. Therefore, a deeper insight into the mechanisms of BMSC osteogenesis will provide new strategies for treating and preventing osteoporosis.

Long non-coding RNAs (lncRNAs) are a class of non-protein-coding RNA transcripts with lengths longer than 200 nucleotides. Growing amounts of evidences have shown that lncRNAs are important regulators in diverse biological processes and diseases including osteoporosis^5–7^. A good number of lncRNAs have been demonstrated to play critical roles in the osteogenic differentiation of BMSCs^8–10^. For example, lncRNA *MALAT1* was downregulated in BMSCs from osteoporosis rats compared with the normal rats, and could inhibit osteogenesis through MAPK signaling^11^.

LncRNA *MIR22HG*, which is located on chromosome 17p13.3, has been discovered to play roles in a variety of cancers. Many previous studies revealed that *MIR22HG* exhibited tumor-suppressive role in several types of cancer including lung cancer, hepatocellular carcinoma, endometrial cancer, gastric cancer and cholangiocarcinoma, and its low expression was associated with poor prognosis^12–17^. Conversely, some studies showed that *MIR22HG* played an oncogenic function in several cancer types such as glioblastoma, and *MIR22HG* knockdown inhibits the invasion and proliferation of cancer cells^18^. However, no study reported the function of *MIR22HG* in the regulation of bone metabolism.

In this study, we established osteoporosis mice model and found *MIR22HG* was significantly downregulated in mouse BMSCs (mBMSCs) from osteoporosis mice compared with the normal mice. *MIR22HG* was increased during the osteogenic differentiation of human BMSCs (hBMSCs) and acted as a positive regulator for hBMSC osteogenesis not only in vitro, but also in vivo. Mechanistically, we demonstrated that *MIR22HG* promoted osteogenic differentiation of hBMSCs via PTEN/AKT signaling. Overall, these findings suggested that *MIR22HG* might serve as a promising therapeutic target for osteoporosis treatment and prevention.

## Materials and methods

### Cell cultivation

Primary hBMSCs, human adipose-derived stem cells (hASCs) and RAW264.7 cells were purchased from ScienCell company (Carlsbad, CA, USA). Cells were cultured in proliferation medium (PM) consisting of DMEM supplemented with 10% fetal bovine serum and 1% antibiotics. For osteogenic differentiation, hBMSCs and hASCs were induced in osteogenic media (OM) composed of standard PM supplemented with 100 nM dexamethasone, 0.2 mM ascorbic acid, and 10 mM β-glycerophosphate. All cell-based in vitro experiments were performed at least three times.

### Transfection

The recombinant lentiviruses containing full-length *MIR22HG* and the scramble control (NC) were purchased from Cyagen Biosciences (Guangzhou, China). Recombinant lentiviruses targeting *MIR22HG* (sh*MIR22HG*-1 and sh*MIR22HG*-2) and the non-targeting scramble control (shNC) were obtained from GenePharma Co. (Shanghai, China). The plasmid pcDNA3.1(+)-PTEN and pcDNA3.1(+), small interfering RNAs targeting PTEN (si-PTEN) and the scramble control (si-NC) were obtained from Integrated Biotech Solutions Co. (Shanghai, China). The sequences are listed in Supplemental Table 1.

### Alkaline phosphatase (ALP) staining and activity

ALP staining and ALP activity assay were performed as described previously^19^. 7 days after osteogenic induction, cells were rinsed with PBS, fixed with 4% paraformaldehyde, incubated with the NBT/BCIP staining kit (CoWin Biotech, Beijing, China). ALP activity was assayed with the ALP Activity Kit (Biovision, Milpitas, CA) and normalized to the total protein contents.

### Alizarin red S staining and quantification

14 days after osteogenic induction, cells were washed with PBS, fixed with 4% paraformaldehyde, and stained with 1% Alizarin red S solution for 20 min. To quantify the degree of mineralization of hBMSCs, the stain was solubilized by cetylpyridinium chloride and quantified by a spectrophotometer at 570 nm.

### RNA isolation and quantitative reverse transcription-polymerase chain reaction (qRT-PCR) analysis

Total RNA was extracted from cells with TRIzol Reagent (Invitrogen, Carlsbad, CA, USA). Then the RNA was reverse transcribed into cDNA using PrimeScript RT Reagent Kit (Takara, Tokyo, Japan). qRT-PCR was amplified with SYBR Green Master Mix (Roche Applied Science, Mannheim, Germany) on a 7500 Real-Time PCR Detection system (Applied Biosystems, Foster City, CA, USA). The comparative cycle threshold (CT) method was used to calculate the relative gene expression. The primers used for Bulge-loop reverse-transcription PCR for microRNA-22 (miR-22) and U6 were purchased from RiboBio (Guangzhou, China). The sequences of primers used are listed in Supplemental Table 1.

### Western blot analysis

Cells were lysed using radioimmunoprecipitation assay (RIPA) lysis buffer. Samples were separated by electrophoresis and then transferred to PVDF membranes (Millipore, Billerica, MA, USA). The membranes were blocked with skimmed milk and incubated with primary antibodies against AKT (Cell Signaling Technology, Beverly, MA, USA), phosphorylated-AKT (Ser473) (Cell Signaling Technology), PTEN (Cell Signaling Technology) and GAPDH (HuaxingBio Science, Beijing, China) at 4 °C overnight. Then, the membranes were incubated with secondary antibodies for 1 h at room temperature. Signals were visualized using the ECL Kit (CoWin Biotech).

### Establishment of ovariectomized mouse model

All animal experiments procedures were performed in compliance with the technical guidelines and approved by the Peking University Animal Care and Use Committee. Twenty BALB/c female mice (8 weeks, 18–20 g) were purchased from Vital Co. (Beijing, China) and randomly divided to two groups: the ovariectomized (OVX) group, non-OVX control (SHAM) group. The mice in the OVX group received bilateral ovariectomy surgery. As a control, the mice in the SHAM group were subjected to removal of adipose tissue near the ovaries. After 6 weeks, mice were sacrificed under anesthesia and the femurs or tibias were collected for the related assays.

mBMSCs were isolated and purified from the tibias of SHAM and OVX mice as described previously^20^. Flow cytometry analysis sorting was performed to screen mBMSCs which were positive for CD44 and CD29 but negative for CD45. The multilineage differentiation potential of isolated mBMSCs was evaluated via osteogenic differentiation and adipogenic differentiation. For osteogenic differentiation, mBMSCs were induced in OM for 7 days and ALP staining was performed. For adipogenic differentiation, mBMSCs were cultured in standard PM supplemented with 50 nM insulin, 100 nM dexamethasone, 0.5 mM 3- isobutyl-1-methylxanthine, and 200 mM indomethacin for 14 days. Oil red O staining was performed as described before^21^.

### Micro CT analyses of mice

Six weeks after the ovariectomy surgery, femurs were harvested and scanned using a Micro CT system (pixel size 8.82 mm, working voltage 80 kV, working current 500 mA, exposure time 1500 ms). Three-dimensional reconstruction was performed with multimodal 3D visualization software (Inveon, Siemens, Munich, Germany). Bone volume/total volume (BV/TV), trabecular number (Tb.N), and trabecular spacing (Tb.Sp) in the trabecular region (1.5 mm distal to the proximal epiphysis) were analyzed using an Inveon Research Workplace (Siemens). Then the femurs were decalcified in 10% ethylene diamine tetraacetic acid (EDTA) and stained with hematoxylin and eosin (HE).

### Ectopic bone formation in vivo

The hBMSCs infected with lentivirus were induced in OM for 7 days before the in vivo experiments. Next, 5 × 10^6^ cells were mixed with 7 mm × 5 mm × 2 mm Bio-Oss Collagen (Geistlich, GEWO GmbH, Baden-Baden, Germany), incubated for 1 h at 37 °C, and then implanted subcutaneously into the dorsal surface of 8-week-old BALB/c homozygous nude (nu/nu) female mice (*n* = 6 per group). The mice were randomized into 5 groups: shNC-hBMSCs/Collagen group, sh*MIR22HG*-1-hBMSCs/Collagen group, sh*MIR22HG*-2-hBMSCs/Collagen group, NC-hBMSCs/Collagen group and *MIR22HG*-hBMSCs/Collagen group. The implants were collected after 8 weeks and fixed with 4% formalin, decalcified, and embedded in paraffin wax. Sections (5 μm) were cut and stained with HE, Masson’s trichrome and immunohistochemical analysis. Immunohistochemical analysis was performed to detect the expression level of osteocalcin (OCN) (anti-OCN, Cell Signaling Technology).

### RNA sequencing

Total RNA isolated from *MIR22HG* knockdown or shNC hBMSCs was used as input material for the RNA sample preparations. cDNA was synthesized and then PCR was performed with phusion high-fidelity DNA polymerase, universal PCR primers and index primer. Finally, PCR products were purified (AMPure XP system) and library quality was evaluated on the Agilent Bioanalyzer 2100 system. Thereafter, the clustering of the index-coded samples was performed on a cBot Cluster Generation System using TruSeq PE Cluster Kit v3-cBot-HS (Illumia). After cluster generation, the library preparations were sequenced on an Illumina NovaSeq platform and 150 bp paired-end reads were generated. Differentially expressed genes were defined as those with a *P* value < 0.05 and a Fold change >2.

### Fluorescent in situ hybridization (FISH)

FISH was conducted with a Fluorescent In Situ Hybridization Kit (RiboBio). Briefly, cells were washed in PBS, fixed with 4% formaldehyde, and then permeabilized in PBS containing 0.5% Triton X-100 at 4 °C for 5 min. Subsequently, cells were prehybridizated at 37 °C for 30 min and then hybridized with anti-*MIR22HG*, anti-U6, or anti-18S oligodeoxynucleotide probe at 37 °C overnight. Then the cells were stained with DAPI for detection of nuclei and captured using a confocal microscope (Carl Zeiss, Germany).

### Subcellular fractionation

Nuclear and cytoplasmic fractions of hBMSCs were separated using a Nuclei Isolation Kit (Invent-biotech, Minnesota, USA). RNA was isolated from both fractions with Trizol and then reverse transcribed into cDNA. The RNA expression was quantified by qRT-PCR as described above^19^. *MALAT1* and *GAPDH* were used as fractionation indicators. The primers used are listed in Supplementary Table 1.

### Colocalization of *MIR22HG* and PTEN

The colocalization of *MIR22HG* and PTEN was performed as previously described^22^. After hybridization with an anti-*MIR22HG* probe (RiboBio), hBMSCs were washed with PBS and incubated with the PTEN antibody at 4 °C overnight. The next day, the cells were incubated in the dark using a Dylight 488-conjugated secondary antibody (Abbkine, California, USA) and stained with DAPI.

### Tartrate resistant acid phosphatase (TRAP) staining

For osteoclast differentiation, RAW264.7 cells were treated with murine receptor activator of nuclear factor-κB ligand (RANKL) (50 ng/ml, Peprotech, NJ, USA) for 5 days. TRAP staining was performed using an acid phosphatase kit (Sigma-Aldrich, St. Louis, MO, USA). Images of TRAP-positive multinucleated cells (containing ≥3 nuclei/cell) were recorded with a microscope.

### Statistical analysis

All statistical values were calculated using SPSS version 16.0 (SPSS Inc., Chicago, IL, USA). Independent sample *t*-test was used to evaluate statistical differences between two groups, and one-way analysis of variance (ANOVA) was used for data of multiple groups. Results were expressed as mean ± standard deviation. A *p* value less than 0.05 was considered statistically significant.

## Results

### *MIR22HG* was decreased in OVX mice

**Fig. 1:**
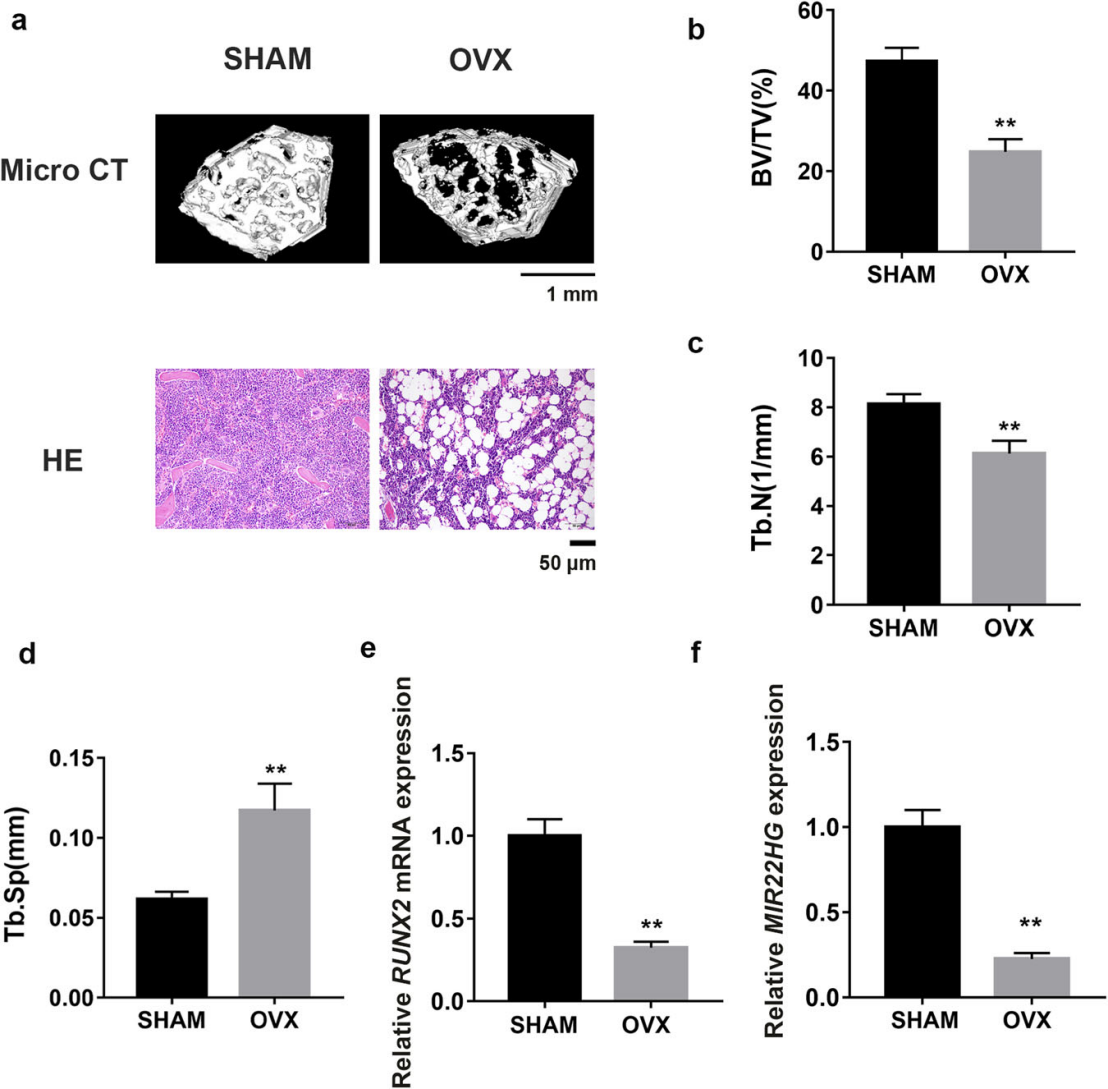
*MIR22HG* expression was decreased in OVX mice. **a** Representative images of Micro CT and HE staining from SHAM and OVX mice indicated the bone loss of OVX mice compared to SHAM mice. Scale bars for Micro CT and HE staining represent 1 mm and 50 μm, respectively. **b**–**d** Trabecular bone volume/tissue volume (BV/TV), trabecular number (Tb.N), and trabecular spacing (Tb.Sp) were detected in SHAM and OVX mice. **e**, **f** Expression levels of *RUNX2* and *MIR22HG* tested in mBMSCs from OVX mice in contrast to SHAM mice, determined by qRT-qPCR. All data are shown as mean ± SD, **P < 0.01, compared with SHAM group.

Recent studies indicated that osteoporosis may be caused by abnormal osteogenic differentiation of BMSCs. To investigate the pathological mechanisms of osteoporosis, we constructed osteoporotic model by ovariectomy. HE staining, Micro CT images and analyses showed that the bone trabeculae of OVX mice was remarkably decreased compared with SHAM mice (Fig. 1a–d). At the same time, mBMSCs from both SHAM and OVX mice were isolated, sorted by flow cytometry and determined with differentiation assays (Supplementary Fig. 1A–C). Based on our previous work, expression levels of several interesting genes were chosen to be measured by qRT-PCR and we found many genes were differentially expressed after ovariectomy. Among them, *MIR22HG* appeared to be one of the most differentially expressed genes. As shown in Fig. 1f, the *MIR22HG* expression level was significantly decreased in OVX mice, meanwhile the expression level of *RUNX2*, an osteogenic marker, was also significantly decreased in mBMSCs from OVX mice (Fig. 1e). These results suggested the potential involvement of *MIR22HG* in the compromised osteogenesis of osteoporotic BMSCs.

### *MIR22HG* was increased during osteogenesis of hBMSCs

**Fig. 2:**
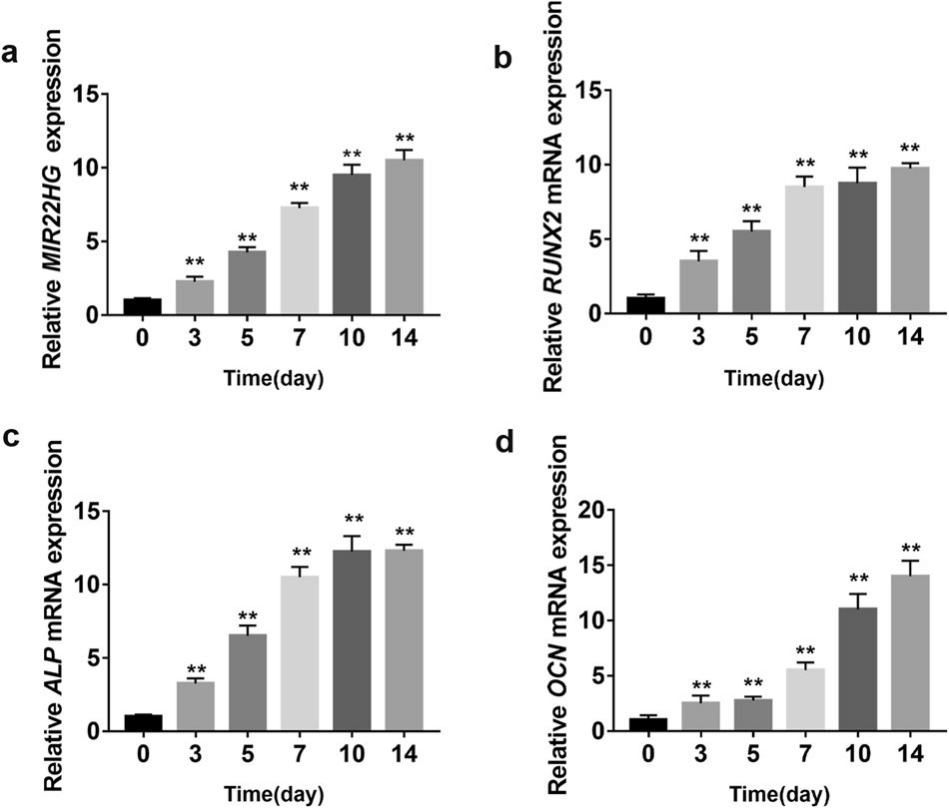
*MIR22HG* was increased during the osteogenic differentiation of hBMSCs. **a** qRT-PCR analysis of *MIR22HG* during the osteogenic differentiation of hBMSCs. **b**–**d** Relative mRNA expression levels of *RUNX2*, *ALP*, and *OCN* were detected by qRT-PCR. Results are presented as the mean ± SD, **P < 0.01, normalized by Glyceraldehyde 3-phosphate dehydrogenase (*GAPDH*), compared with day 0.

To elucidate whether *MIR22HG* plays a role in osteogenic differentiation of BMSCs, we first attempted to profile *MIR22HG* expression pattern in hBMSCs after osteogenic induction. qRT-PCR result indicated that the expression of *MIR22HG* was significantly upregulated (Fig. 2a). As expected, the mRNA levels of osteogenesis-related genes *RUNX2*, *ALP,* and *OCN* were significantly increased (Fig. 2b–d).

### *MIR22HG* promoted osteogenic differentiation

**Fig. 3:**
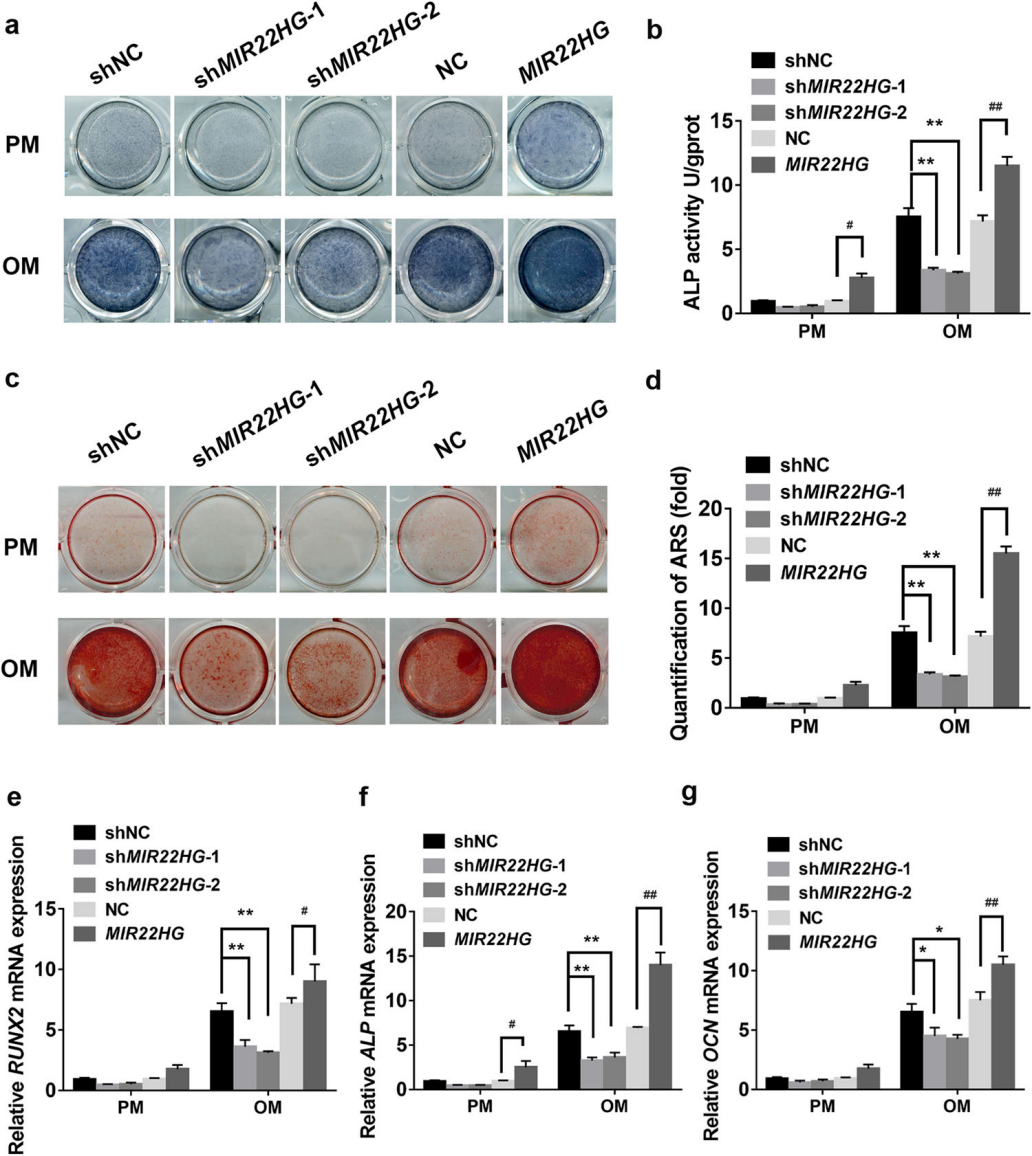
The role of *MIR22HG* in osteogenic differentiation of hBMSCs. **a** Images of ALP staining in shNC, sh*MIR22HG*-1, sh*MIR22HG*-2, NC, *MIR22HG* groups. Cells were treated with proliferation medium (PM) or osteogenic medium (OM) for 7 days. **b** Histogram showing 7d ALP activity. **c**, **d** Alizarin Red S (ARS) staining and quantification in shNC, sh*MIR22HG*-1, sh*MIR22HG*-2, NC, *MIR22HG* groups on day 14. **e**–**g** Relative mRNA expression levels of *RUNX2*, *ALP,* and *OCN* measured by qRT-PCR on day 14 of osteogenic induction. *GAPDH* was used for normalization. Results are presented as the mean ± SD, */^#^*p* < 0.05, **/^##^*p* < 0.01, * compared with shNC, ^#^ compared with NC.

To evaluate the function of *MIR22HG* in the osteogenic differentiation of hBMSCs, we used lentivirus to knockdown or overexpress *MIR22HG* in hBMSCs (Supplementary Fig. 2A–C). Seven days after culturing the transfected cells in PM or OM, ALP activity was significantly increased in the *MIR22HG* overexpression group but decreased in the *MIR22HG* knockdown group (Fig. 3a, b). Consistently, the mineralized nodules, as detected by ARS staining and quantification on day 14, was increased in the *MIR22HG* overexpression group but reduced in the *MIR22HG* knockdown group (Fig. 3c, d). Moreover, the mRNA levels of *RUNX2*, *ALP*, and *OCN* were decreased in the *MIR22HG* knockdown group but upregulated in *MIR22HG* overexpression group (Fig. 3e–g).

Additionally, we also investigated the effect of *MIR22HG* on osteogenesis of hASCs. Results showed *MIR22HG* played a similar function in hASCs (Supplementary Fig. 3A–F). All of these results revealed a positive role of *MIR22HG* in regulating osteogenesis.

### *MIR22HG* promoted bone formation of hBMSCs in vivo

**Fig. 4:**
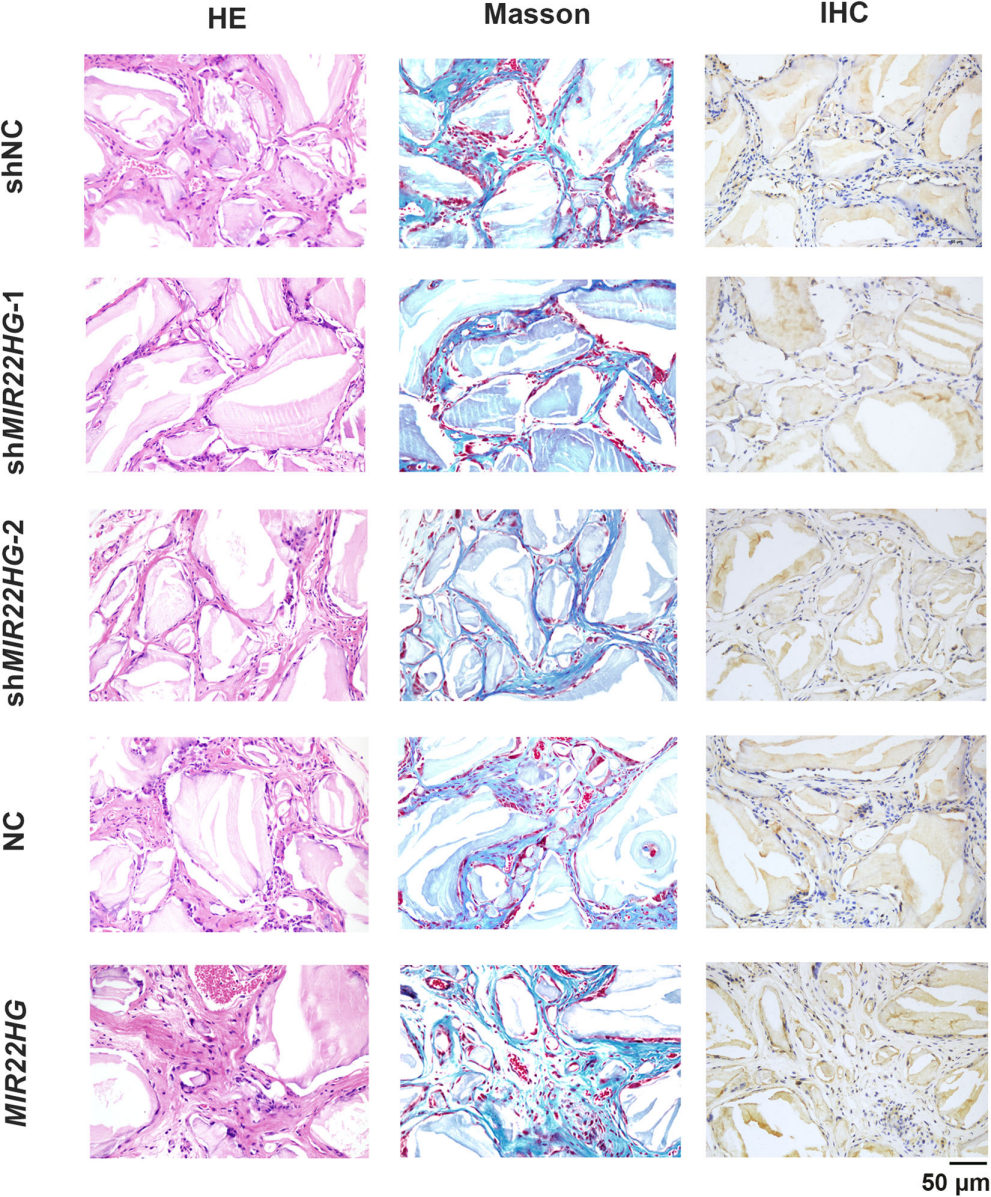
*MIR22HG* promoted bone formation of hBMSCs in vivo. HE staining (HE), Masson’s trichrome staining (Masson), and immunohistochemical staining (IHC) of osteocalcin (OCN) in shNC, sh*MIR22HG*-1, sh*MIR22HG*-2, NC, *MIR22HG* groups. Scale bar = 50 μm.

To further discover the role of *MIR22HG* in osteogenesis, we next tested whether *MIR22HG* could regulate bone formation potential of hBMSCs in vivo. HE staining and Masson’s trichrome staining showed *MIR22HG* overexpression group formed more bone-like tissues compared with its control group (NC), whereas *MIR22HG* knockdown dramatically inhibited the efficiency of new bone formation compared with the control group (shNC). Moreover, we found the expression level of OCN was higher in *MIR22HG* overexpression group but lower in *MIR22HG* knockdown group as revealed by immunohistochemical staining (Fig. 4).

### Differentially expressed genes in *MIR22HG* knockdown BMSCs

**Fig. 5:**
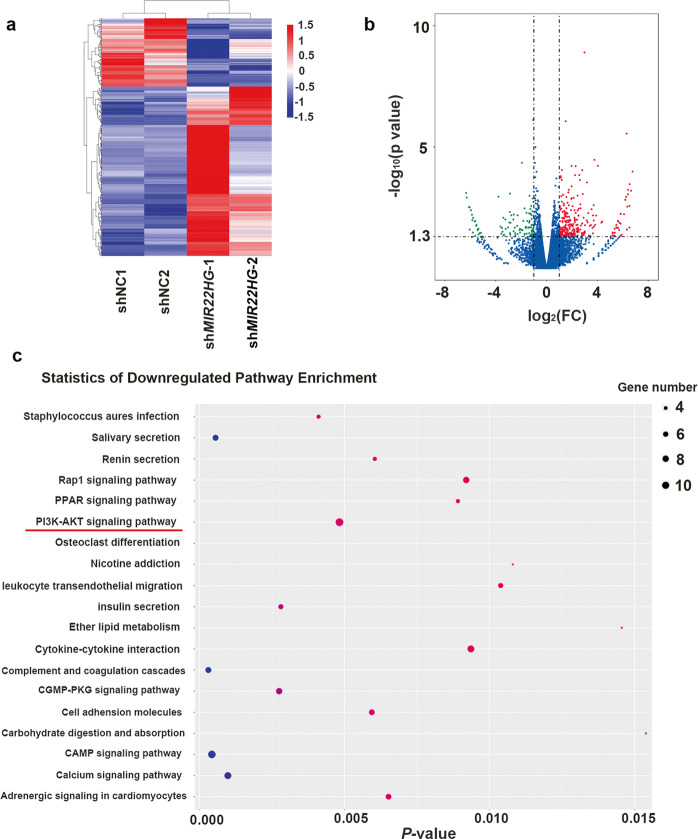
Differential expression of genes between *MIR22HG* knockdown and shNC hBMSCs. **a** The differentially expressed genes in *MIR22HG* knockdown hBMSCs were shown in the heat map. *p* value <0.05 and fold change >2 were set as restrictive conditions to identify the differentially expressed genes. **b** The differentially expressed genes were counted; among these genes, a total of 278 genes were upregulated and 112 genes were downregulated. **c** KEGG pathway analysis showed the genes downregulated by *MIR22HG* knockdown might be related to different pathways, among which the top-ranking enriched pathways were shown.

To further investigate the underlying mechanisms of *MIR22HG* in the regulation of hBMSC osteogenesis, sh*MIR22HG*-1, sh*MIR22HG*-2, and their negative control (shNC) hBMSCs were collected and subjected to RNA sequencing. Heat map revealed the genes that were differentially expressed following *MIR22HG* knockdown (Fig. 5a). Among these genes, 278 genes were upregulated and 112 genes were downregulated (Fig. 5b). Furthermore, KEGG pathway analysis was applied to identify the pathways that were differentially expressed by *MIR22HG* knockdown. Among the downregulated signaling pathways, phosphatidylinositol 3-kinase (PI3K)/AKT signaling which is closely associated with osteogenic differentiation appeared to be one of the most enriched pathways (Fig. 5c).

**Fig. 6:**
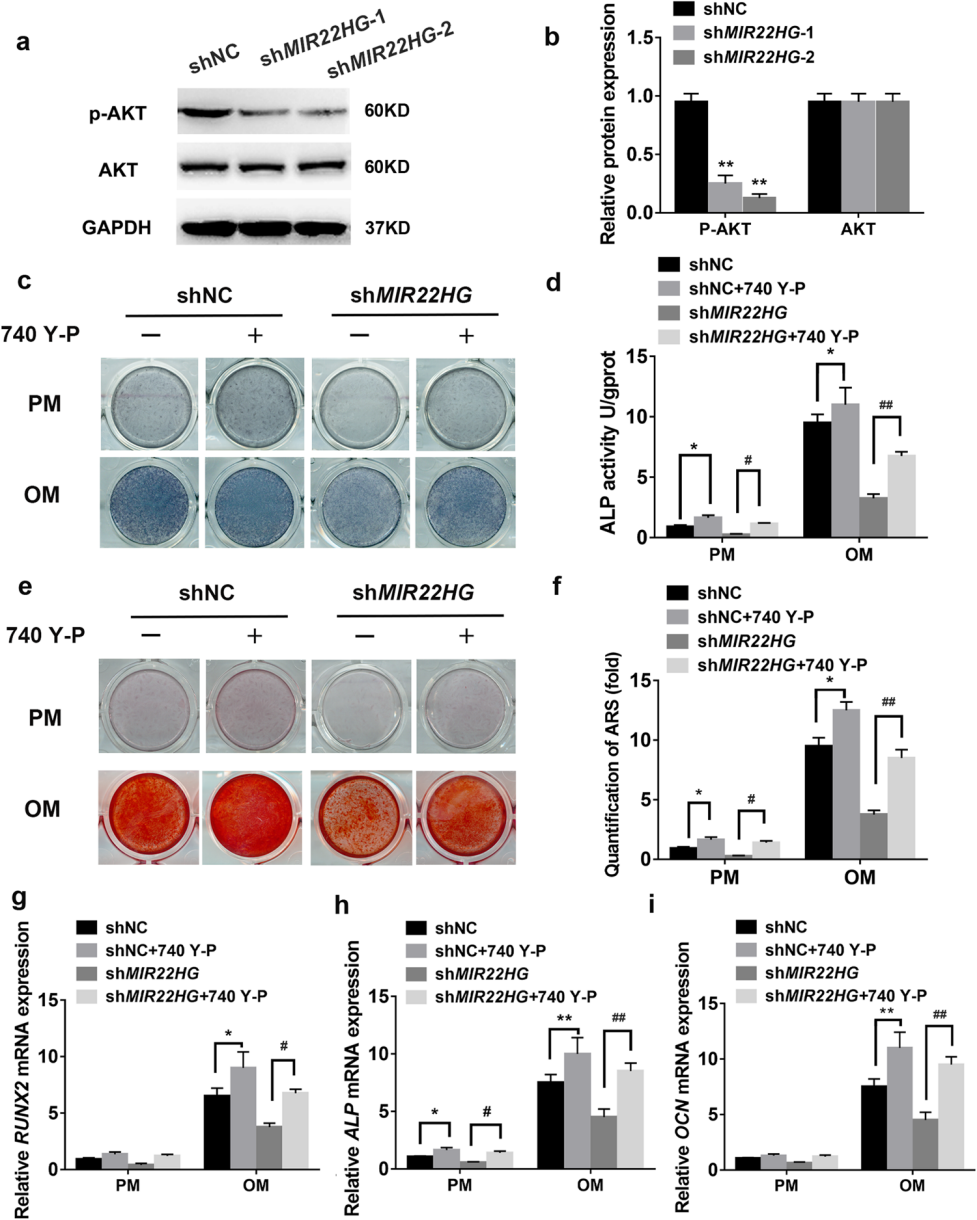
*MIR22HG* knockdown inhibited AKT signaling. **a** The expression levels of total AKT and phosphorylated AKT (p-AKT) in shNC, sh*MIR22HG*-1, and sh*MIR22HG*-2 groups. GAPDH was used as an internal control. **b** The band intensities of **a** were analyzed by Image J software. **c** *MIR22HG* knockdown (sh*MIR22HG*) and the control (shNC) hBMSCs were treated with proliferation or osteogenic media for 7 days. 740 Y-P (10 μM) or DMSO (control ‘-’) was added to the medium for 7 days and ALP staining was performed. **d** Histogram showing 7d ALP activity. **e** Images of Alizarin red S staining (ARS) in shNC, sh*MIR22HG* groups treated with 740 Y-P (10 μM) or DMSO (control ‘-’) for 14 days. **f** Histograms showing quantification of ARS by spectrophotometry. **g**–**i** Relative mRNA expression levels of *RUNX2*, *ALP*, and *OCN* on day 14 after osteogenic induction. 740 Y-P (10 μM) was incubated for 14 days. DMSO was used as control. Results are presented as the mean ± SD, */^#^*p* < 0.05, **/^##^*p* < 0.01, * compared with shNC, ^#^ compared with sh*MIR22HG*.

Furthermore, Western blot showed the level of p-AKT was significantly decreased in the sh*MIR22HG* hBMSCs (Fig. 6a, b), which was consistent with the result of RNA sequencing. We therefore hypothesized that *MIR22HG* might promote the osteogenic differentiation of hBMSCs with the involvement of AKT signaling.

### *MIR22HG* knockdown impaired osteogenesis via inhibiting AKT activation

To determine whether *MIR22HG* knockdown inhibits hBMSC osteogenesis through inhibiting AKT signaling, we treated *MIR22HG* knockdown hBMSCs and the negative control group (shNC) with AKT signaling activator 740 Y-P. Western blot showed 740 Y-P significantly upregulated the level of p-AKT (Supplementary Fig. 4A, B). We next examined whether the addition of 740 Y-P could rescue the impaired osteogenesis caused by *MIR22HG* knockdown. ALP staining and activity showed the administration of 740 Y-P counteracted the impairments of *MIR22HG* knockdown on ALP activity (Fig. 6c, d). Similarly, ARS staining and quantification showed 740 Y-P treatment partially abrogated the inhibitory effect of *MIR22HG* knockdown on mineralization (Fig. 6e, f). Moreover, qRT-PCR analysis revealed the addition of 740 Y-P upregulated *RUNX2*, *ALP*, and *OCN* mRNA levels in sh*MIR22HG* hBMSCs (Fig. 6g–i).

### *MIR22HG* overexpression promoted osteogenesis by activating AKT signaling

**Fig. 7:**
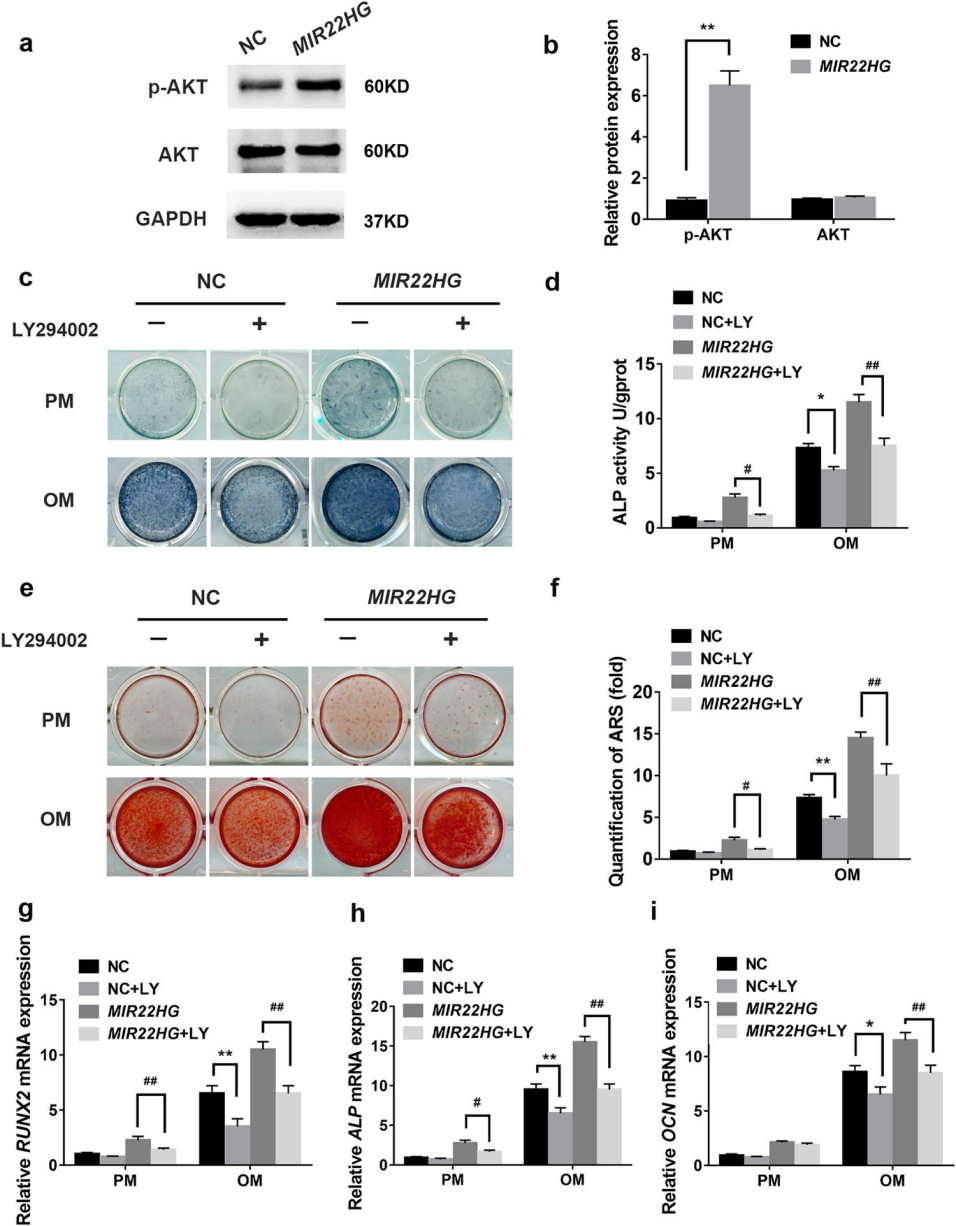
*MIR22HG* overexpression activated AKT signaling. **a** The expression levels of total AKT and phosphorylated AKT (p-AKT) in NC, *MIR22HG* groups. GAPDH was used as an internal control. **b** The band intensities of **a** were analyzed by Image J software. **c** ALP staining in *MIR22HG* overexpression (*MIR22HG*) and the control (NC) hBMSCs with or without LY294002 (10 μM) treatment on day 7 of osteogenic induction. DMSO was used as control (-). **d** Histogram showing 7d ALP activity. **e** Calcium deposition in NC, *MIR22HG* groups treated with LY294002 (10 μM) or DMSO (control ‘-’) was observed by Alizarin Red S staining on day 14 of osteogenic induction. **f** Histograms showing quantification of ARS by spectrophotometry. **g**–**i** Relative mRNA expression of *RUNX2*, *ALP*, and *OCN* on day 14 after osteogenic induction. LY294002 (10 μM) was incubated for 14 days. DMSO was used as control. Results are presented as the mean ± SD, */^#^*p* < 0.05, **/^##^*p* < 0.01, * compared with NC, ^#^ compared with *MIR22HG*. LY: LY294002.

To further verify the relationship between *MIR22HG* and AKT pathway, we also investigated AKT signaling in *MIR22HG* overexpressed hBMSCs. Western blot results showed the level of p-AKT was significantly increased in *MIR22HG* overexpression group compared with NC group (Fig. 7a, b). Next, we treated *MIR22HG* overexpressed hBMSCs and its control group (NC) with PI3K/AKT inhibitor LY294002. It was found that the level of p-AKT was decreased when cells were treated with LY294002 (Supplementary Fig. 4C, D). As shown in Fig. 7c–i, inhibition of PI3K/AKT signaling pathway by LY294002 attenuated the enhancing effect of *MIR22HG* overexpression on osteogenesis.

### The role of PTEN in *MIR22HG*-mediated osteogenesis

Previous studies demonstrated that PTEN inhibits the activation of downstream proteins of AKT signaling^23^, we hypothesized that PTEN played a role in the *MIR22HG*-mediated osteogenesis. Therefore, we next performed Western blot analysis of PTEN in *MIR22HG* knockdown or overexpressed hBMSCs. We found that *MIR22HG* knockdown significantly increased the level of PTEN and decreased the level of p-AKT, whereas *MIR22HG* overexpression had an opposite effect (Supplementary Fig. 5A, C). To determine the effects of PTEN in *MIR22HG*-mediated osteogenesis, we transfected sh*MIR22HG* hBMSCs and the control group (shNC) with si-PTEN. As shown in Supplementary Fig. 5B, the suppression of PTEN blocked the inhibitory effect of *MIR22HG* knockdown on ALP activity. In addition, PTEN overexpression plasmid and the empty control plasmid (PC) were introduced into *MIR22HG* overexpression hBMSCs and its control group (NC). ALP staining showed the increased ALP activity induced by *MIR22HG* overexpression was effectively reversed in the *MIR22HG* and *PTEN* double overexpressed hBMSCs (Supplementary Fig. 5D). These results suggested *MIR22HG* regulates osteogenic differentiation of hBMSCs via PTEN/AKT signaling.

### The distribution of *MIR22HG*

Since the function of most lncRNAs is closely related to their subcellular location^24^, we also investigated the distribution of *MIR22HG* of hBMSCs by FISH assay. As shown in Supplementary Fig. 6A, *MIR22HG* was mainly distributed in the nucleus of hBMSCs. Consistently, subcellular fractionation assay also demonstrated that *MIR22HG* was primarily located in the nucleus, meanwhile the marker RNAs (*MALAT1* and *GAPDH*) were enriched in their expected fractions (Supplementary Fig. 6B). Moreover, confocal microscopy for lncRNA *MIR22HG* FISH and PTEN immunostaining revealed that *MIR22HG* was localized predominantly in the nucleus, while PTEN was localized both in the cytoplasm and nucleus (Supplementary Fig. 6C). PTEN has been shown to play various roles in different cellular compartments. Nuclear PTEN is essential for maintaining chromosomal integrity and genomic stability. Cytoplasmic PTEN converts phosphatidylinositol-3,4,5-trisphosphate (PIP3) to phosphatidylinositol-4,5-bisphosphate (PIP2), thereby repressing PI3K/AKT pathway^25^. It was reported that miR-22 reduced the level of PTEN via directly combining to the 3’UTR of PTEN^26^. Thus, it was reasonable to hypothesize that *MIR22HG* regulated the level of PTEN through miR-22. As shown in Supplementary Fig. 6D, the level of miR-22 was significantly increased in *MIR22HG* overexpression hBMSCs. Collectively, these results indicated that *MIR22HG* might regulate PTEN through miR-22.

### The role of *MIR22HG* in osteoclast differentiation

It has been demonstrated that the imbalance between osteoblastic bone formation and osteoclastic bone resorption can cause osteoporosis^27^. Understanding the mechanisms of osteoclastogenesis is essential to develop treatments for osteoporosis. Therefore, we also investigated the role of *MIR22HG* in osteoclast differentiation. We found the expression level of *MIR22HG* was increased during RANKL-induced osteoclastogenesis of RAW264.7 cells (Supplementary Fig. 7A). Gain- and loss-of-functions studies showed *MIR22HG* promoted osteoclast differentiation of RAW264.7 cells (Supplementary Fig. 7B–E).

## Discussion

Recent studies have reported that lncRNAs could act as key regulators of osteogenic differentiation and pathological processes of osteoporosis^28^. Fei et al. reported that 51 lncRNAs were differentially expressed in patients with osteoporosis compared with healthy controls^29^. Shen et al. showed that the expression of lncRNA *HOTAIR* in osteoporosis patients was significantly higher than that in normal people. They found *HOTAIR* suppressed osteogenic differentiation of rat BMSCs through inhibiting the Wnt/β-catenin pathway^30^. Yang et al. demonstrated that lncRNA *ORLNC1* alleviated osteoporosis by enhancing osteogenesis through the increase of BMP2 by sponging miR-140–5p^31^.

In our research, we examined the relative level of *MIR22HG* in mouse model of osteoporosis and found the expression of *MIR22HG* was lower compared with SHAM mice. We observed that *MIR22HG* was upregulated during the osteogenic differentiation of hBMSCs. Thus, we investigated the role of *MIR22HG* in regulating osteogenic differentiation. We found *MIR22HG* knockdown significantly inhibited osteogenesis, while *MIR22HG* overexpression enhanced osteogenesis of hBMSCs both in vitro and in vivo. These results implied the positive effect of *MIR22HG* in osteogenic differentiation.

Various studies have provided evidence that the PI3K/AKT signaling plays an important role in regulating bone development. PI3K/AKT is negatively modulated by PTEN^32^. The loss of PTEN function in osteoblasts leads to persistent AKT activation, and subsequently increased osteogenic differentiation^33^. Previous studies discovered that several lncRNAs interacted with AKT pathway in the process of osteogenic differentiation. For example, Wu et al. showed that lncRNA *HIF1A-AS2* promoted osteogenic differentiation of adipose-derived stem cells through activating the PI3K/AKT signaling pathway^34^. Zhang et al. reported that AKT signaling was involved in lncRNA *NKILA*-mediated osteogenic differentiation in mesenchymal stem cells^35^.

In the current research, we found *MIR22HG* overexpression increased p-AKT expression, and the enhancing effect of *MIR22HG* overexpression on osteogenesis was abrogated by LY294002 or pcDNA3.1(+)-PTEN transfection. Moreover, *MIR22HG* knockdown inhibited osteogenesis of hBMSCs and downregulated the phosphorylation level of AKT. The addition of 740 Y-P or si-PTEN reversed the inhibitory effect of *MIR22HG* knockdown on osteogenesis of hBMSCs. These results revealed that *MIR22HG* affected osteogenic differentiation of hBMSCs by modulating PTEN/AKT pathway.

Studies have shown that the intracellular distribution of lncRNAs is associated with their function and potential molecular roles^36^. Thus, we detected the subcellular localization of *MIR22HG* by FISH and subcellular fractionation assay. The results all revealed that *MIR22HG* was primarily located in the nucleus of hBMSCs. However, this result was contrary to a previous study, which found that *MIR22HG* was mostly located in the cytoplasm of lung cancer cell lines^12^. This discrepancy may suggest that the role of *MIR22HG* varies between different cell types. To investigate the relationship between distribution of *MIR22HG* and PTEN/AKT pathway, we performed fluorescence colocalization microscopy analysis and observed that *MIR22HG* was mainly distributed in the nucleus while PTEN was presented both in the nucleus and cytoplasm. PTEN has been verified to play various roles in different cellular compartments. Multiple studies have confirmed that cytoplasmic PTEN is an antagonist of the PI3K/AKT pathway because of its lipid phosphatase activity against PIP3. Recently, growing evidence demonstrates that nuclear PTEN is important for maintaining genomic stability through critical roles in modulating DNA repair and cell-cycle arrest^37^. Although several components of the PI3K/AKT pathway also existed in the nucleus, such as PI3K, PIP3 and AKT, cytoplasmic but not nuclear pools of PIP3 have been discovered to be sensitive to the lipid phosphatase activity of PTEN^38^. With the rapid development of high-throughput sequencing, a number of miRNAs were identified to be involved in PTEN downregulation. miR-22 has been proved to downregulate PTEN in many different cell types via directly binding to the 3’UTR of PTEN^39,40^. In this study, we found *MIR22HG* overexpression significantly promoted miR-22 expression in hBMSCs. Based on these results, we speculated that *MIR22HG* might regulate PTEN through miR-22. More experimental studies are needed to further confirm this viewpoint.

Osteoporosis results from an imbalance between osteoblast-mediated bone formation and osteoclast-mediated bone resorption. In this study, we also examined the function of *MIR22HG* in osteoclastogenesis and found *MIR22HG* promoted osteoclast differentiation of RAW264.7 cells. Sugatani et al. reported that PTEN overexpression inhibited RANKL-induced osteoclast differentiation^41^. Moon et al. demonstrated that AKT stimulated osteoclast differentiation via GSK3β/NFATC1 signaling cascade^42^. In this study, we found that *MIR22HG* reduced the level of PTEN and activated AKT pathway. We reasoned that *MIR22HG* might regulate osteoclastogenesis with an involvement of PTEN/AKT pathway. Future studies will try to characterize the potential interactions between *MIR22HG* and these pathways.

In summary, we found for the first time that lncRNA *MIR22HG* was downregulated in the osteoporotic model, and *MIR22HG* overexpression promoted osteogenic differentiation of hBMSCs both in vitro and in vivo. These results suggested that *MIR22HG* might be utilized as a novel diagnostic and therapeutic target for osteoporosis.

## Supplementary information

Supplemental Information

Supplemental Figure 1

Supplemental Figure 2

Supplemental Figure 3

Supplemental Figure 4

Supplemental Figure 5

Supplemental Figure 6

Supplemental Figure 7

Supplemental table 1

## Data Availability

The authors declare that all data supporting the findings of this study are available within the paper and its supplementary information files.
