# Supplementary material for: Long non-coding RNA MIR22HG promotes osteogenic differentiation of bone marrow mesenchymal stem cells via PTEN/ AKT pathway
Source: Cell Death Dis. 2020 Jul 30;11(7):601. doi: 10.1038/s41419-020-02813-2 (PMC7393093; doi:10.1038/s41419-020-02813-2)
Supplement: Supplementary file 1 — Supplemental Information [file 41419_2020_2813_MOESM1_ESM.docx]

**Supplemental Information**

**Supplemental Figure 1** Identification of mBMSCs

(A) Flow cytometry analysis sorting was performed to screen mBMSCs which were positive for CD44 and CD29 but negative for CD45. (B) To induce osteogenic differentiation, mBMSCs were cultured in osteogenic medium (OM). 7 days after culturing, ALP staining was performed. Scale bars: 100 μm. (C) To induce adipogenic differentiation, mBMSCs were cultured in adipogenic medium (AM). At day 7, Oil red o staining was applied to record lipid formation. Scale bars: 100 μm.

**Supplemental Figure 2** Lentivirus transfection to knockdown or overexpress *MIR22HG* in hBMSCs

(A) Microscopic images of GFP-positive hBMSCs under ordinary and fluorescent light. The GFP-positive fluorescent staining showed the efficiency of lentiviral transduction was over 90%. Scale bar = 200 µm. (B) Relative expression of *MIR22HG* in shNC, sh*MIR22HG*-1 and sh*MIR22HG*-2 hBMSCs, as revealed by qRT-PCR. (C) Relative expression of *MIR22HG* in NC, *MIR22HG* hBMSCs, as revealed by qRT-PCR. Results are presented as the mean ± SD, ***P* < 0.01, compared with shNC or NC.

**Supplemental Figure 3** The role of *MIR22HG* in osteogenic differentiation of human adipose derived stem cells (hASCs)

(A) *MIR22HG* was increased during the osteogenic differentiation of hASCs, as shown by qRT-PCR analysis. Results are presented as the mean ± SD, ***P* < 0.01, normalized by Glyceraldehyde 3-phosphate dehydrogenase (*GAPDH*), compared with day 0. (B) Lentivirus transfection was applied to knockdown or overexpress *MIR22HG* in hASCs, and confirmed by qRT-PCR. (C) Cells were treated with proliferation medium (PM) or osteogenic medium (OM). After 7 days, ALP staining was performed and results showed that *MIR22HG* knockdown reduced ALP activity of hASCs, whereas *MIR22HG* overexpression increased ALP activity of hASCs. (D) Histogram showing 7d ALP activity. (E, F) Cells were treated with PM or OM for 14 days. Alizarin Red S staining (ARS) and quantification showed that *MIR22HG* knockdown decreased mineralization of hASCs, whereas *MIR22HG* overexpression increased mineralization of hASCs. Results are presented as the mean ± SD, */**^#^***p* < 0.05, **/**^##^***p* < 0.01，* compared with shNC, **^#^** compared with NC.

**Supplemental Figure 4** The addition of 740 Y-P or LY294002 to hBMSCs

(A) The levels of total AKT and phosphorylated AKT (p-AKT) in shNC and sh*MIR22HG* hBMSCs after incubation with 740 Y-P (10 μM) were assessed using Western blot analysis. DMSO was used as control. (B) The quantitative results of (A) by Image J software. (C) The levels of total AKT and phosphorylated AKT (p-AKT) in NC and *MIR22HG* hBMSCs after incubation with LY294002 (10 μM) determined by Western blot analysis. (D) The quantitative results of (C) by Image J software. GAPDH was used as an internal control. Results are presented as the mean ± SD, **p* < 0.05, **/**^##^***p* < 0.01，* compared with shNC or NC, **^#^** compared with sh*MIR22HG* or *MIR22HG*.

**Supplemental Figure 5** The role of PTEN in *MIR22HG*-regulated osteogenesis

(A) The levels of PTEN, total AKT and phosphorylated AKT (p-AKT) in shNC and sh*MIR22HG* hBMSCs determined by Western blot analysis. (B) Silencing of PTEN with si-PTEN significantly increased the *MIR22HG* knockdown-inhibited ALP activity compared to scrambled siRNA (si-NC), as determined by ALP staining. (C) The levels of PTEN, total AKT and phosphorylated AKT (p-AKT) in NC and *MIR22HG* hBMSCs determined by Western blot analysis. (D) PTEN overexpression through pcDNA3.1(+)-PTEN infection reversed the *MIR22HG* overexpression-enhanced ALP activity compared to empty pcDNA3.1 plasmid (PC).

**Supplemental Figure 6** The distribution of *MIR22HG* in hBMSCs

(A) Fluorescence in Situ Hybridization (FISH) assay used to examine the location of *MIR22HG* in hBMSCs. U6, probe for U6 snRNA, 18S, probe for 18S rRNA. Scale bars: 50 μm. (B) Percentage of nuclear and cytoplasmic RNA levels of *MIR22HG*, *MALAT1*, and *GAPDH* in hBMSCs determined by qRT-PCR. (C) Confocal microscopy images showing colocalization of *MIR22HG* and PTEN in hBMSCs. Scale bars: 50 μm. (D) The expression of miR-22 was increased in *MIR22HG* overexpression hBMSCs, as shown by qRT-PCR analysis. Results are presented as the mean ± SD, ***P* < 0.01, compared with NC.

**Supplemental Figure 7** The role of *MIR22HG* in osteoclast differentiation of RAW264.7 cells

(A) *MIR22HG* was upregulated during the osteoclast differentiation of RAW264.7 cells, as shown by qRT-PCR analysis. Results are presented as the mean ± SD, ***P* < 0.01, normalized by Glyceraldehyde 3-phosphate dehydrogenase (*GAPDH*), compared with day 0. (B) Lentivirus transfection was applied to knockdown or overexpress *MIR22HG* in RAW264.7 cells, and confirmed by qRT-PCR. (C, D) *MIR22HG* knockdown inhibited the mRNA expression of osteoclast marker genes (*TRAP*, *NFATC1*), whereas *MIR22HG* overexpression increased the expression of *TRAP* and *NFATC1*, as determined by qRT-PCR. (E) Cells were cultured with RANKL (50 ng/ml) for 5 days, and TRAP staining was performed. TRAP staining showed *MIR22HG* knockdown significantly reduced the number of RAW264.7-differentiated osteoclasts, whereas *MIR22HG* overexpression group formed more and larger osteoclasts. Scale bars: 50 μm. Results are presented as the mean ± SD, **/**^##^***p* < 0.01，* compared with shNC, **^#^** compared with NC.
