# Supplementary figures and images for: Long non-coding RNA MIR22HG promotes osteogenic differentiation of bone marrow mesenchymal stem cells via PTEN/ AKT pathway
Source: Cell Death Dis. 2020 Jul 30;11(7):601. doi: 10.1038/s41419-020-02813-2 (PMC7393093; doi:10.1038/s41419-020-02813-2)

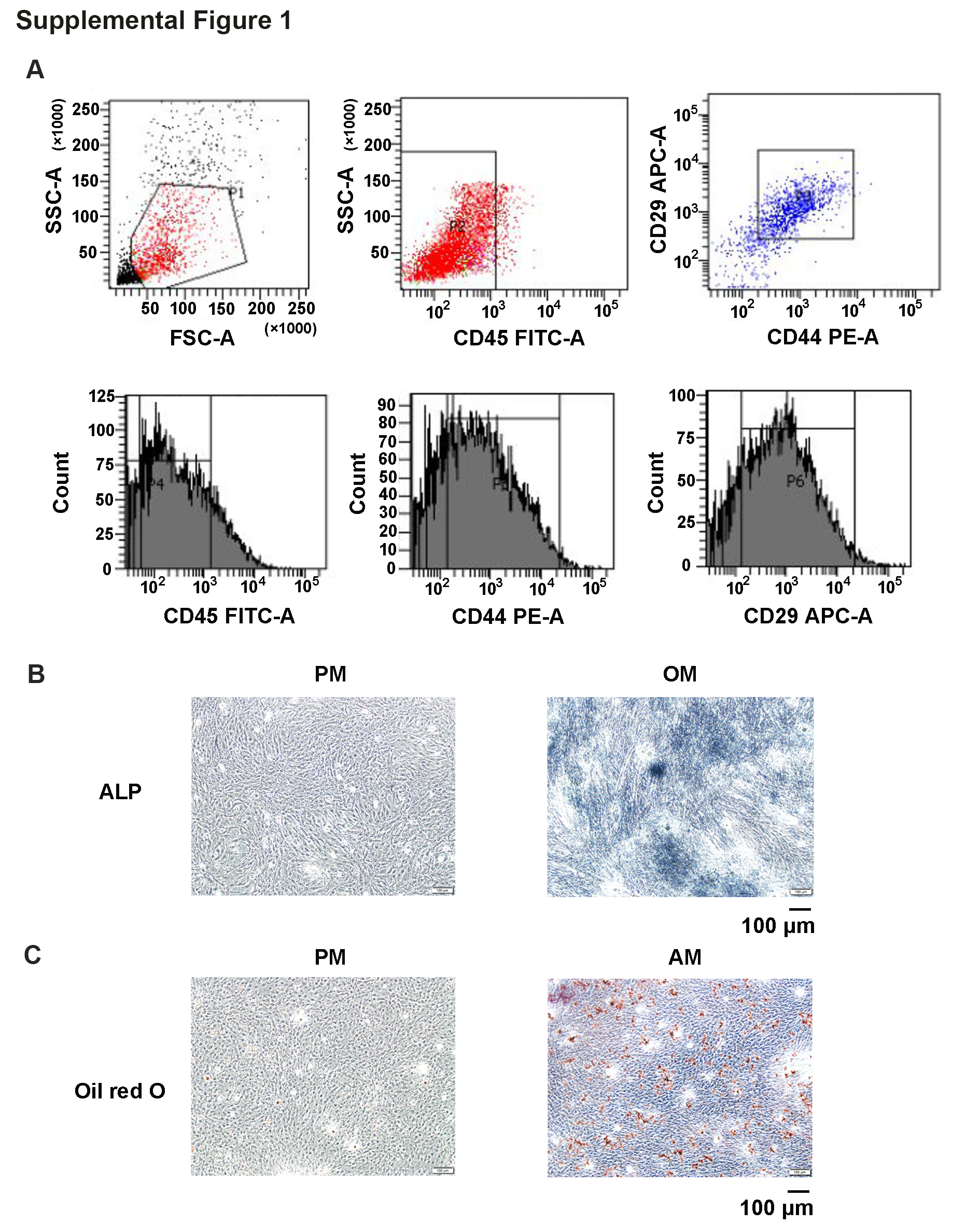

Supplement: Supplementary file 2 — Supplemental Figure 1 [file 41419_2020_2813_MOESM2_ESM.tif]

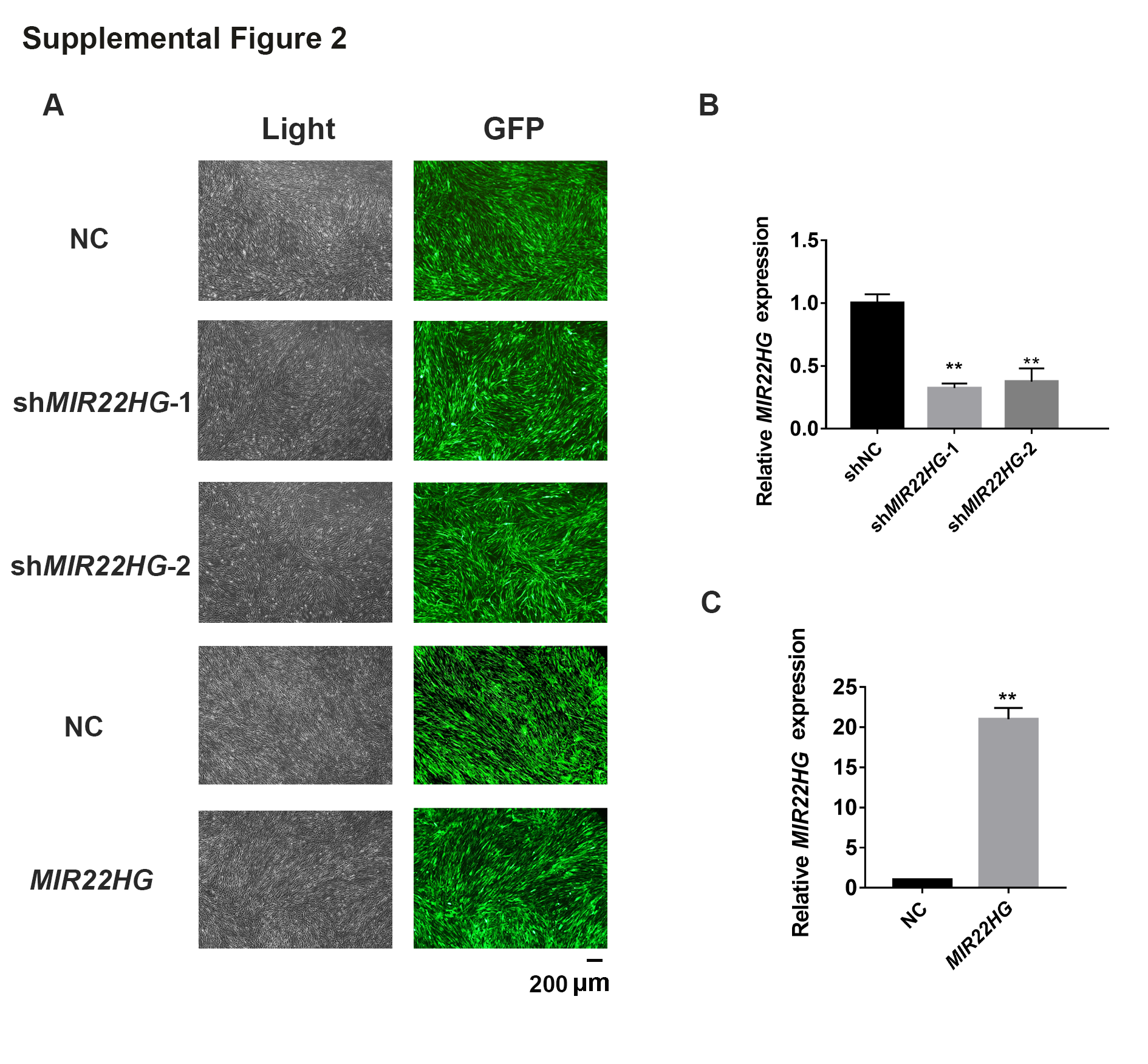

Supplement: Supplementary file 3 — Supplemental Figure 2 [file 41419_2020_2813_MOESM3_ESM.tif]

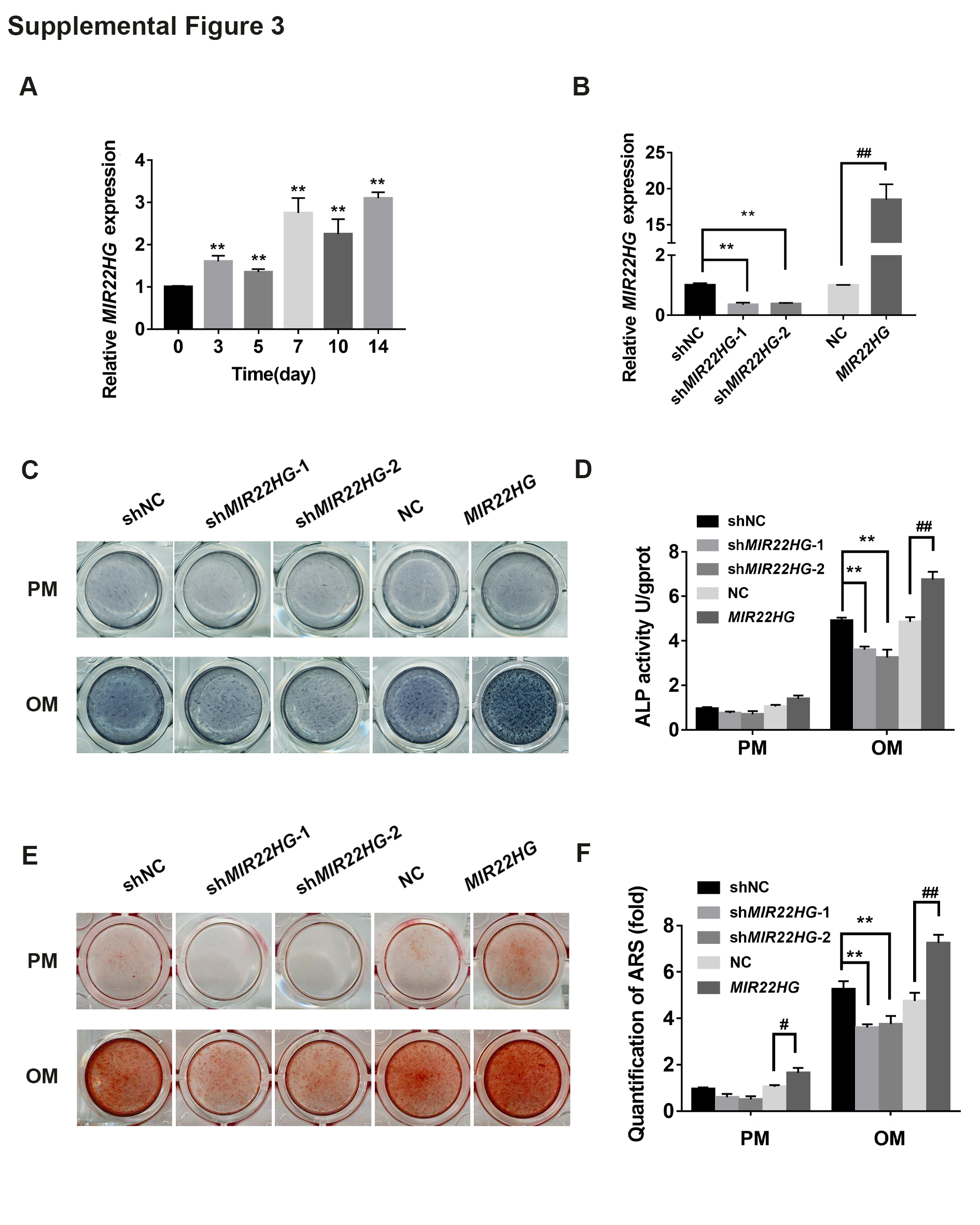

Supplement: Supplementary file 4 — Supplemental Figure 3 [file 41419_2020_2813_MOESM4_ESM.tif]

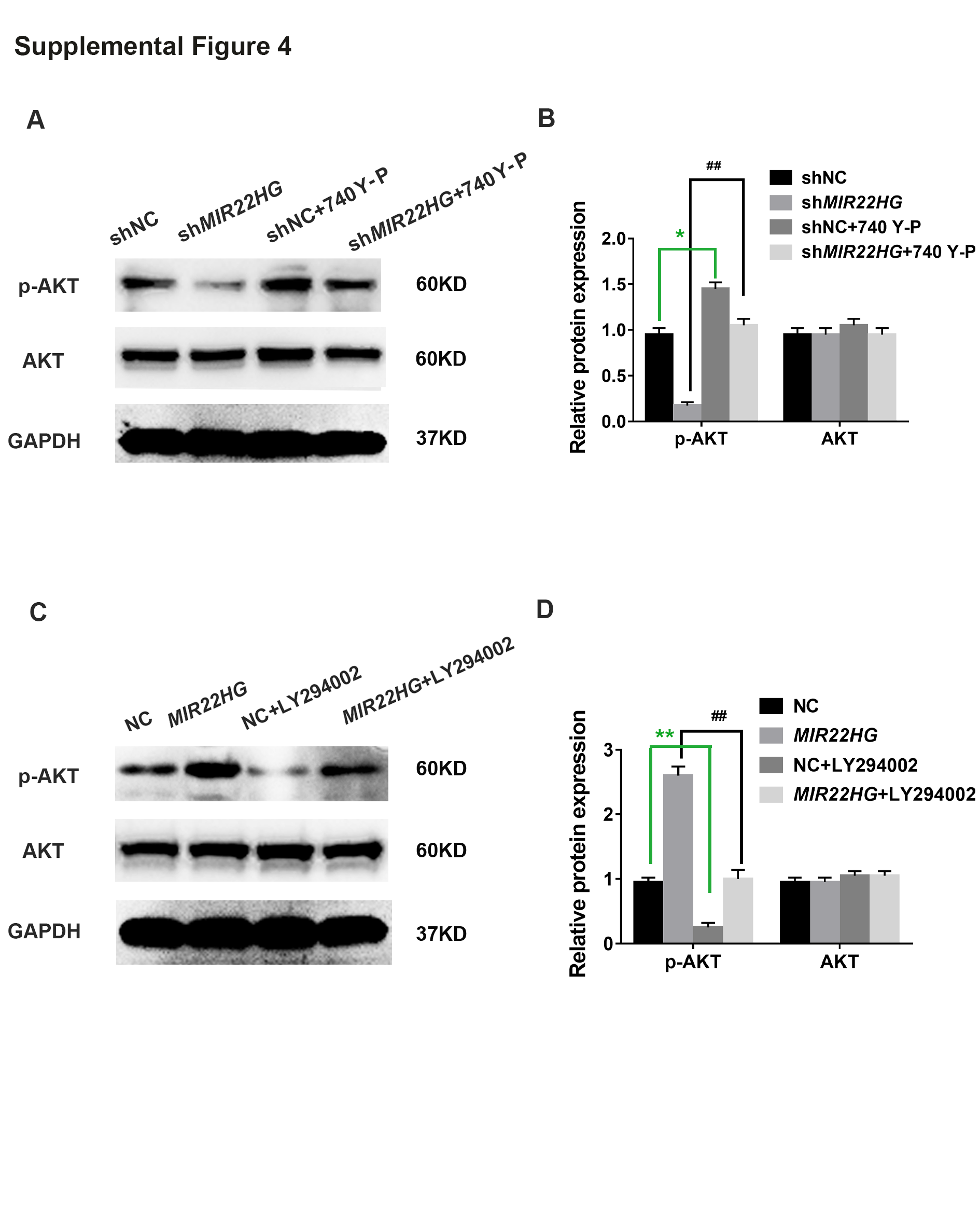

Supplement: Supplementary file 5 — Supplemental Figure 4 [file 41419_2020_2813_MOESM5_ESM.tif]

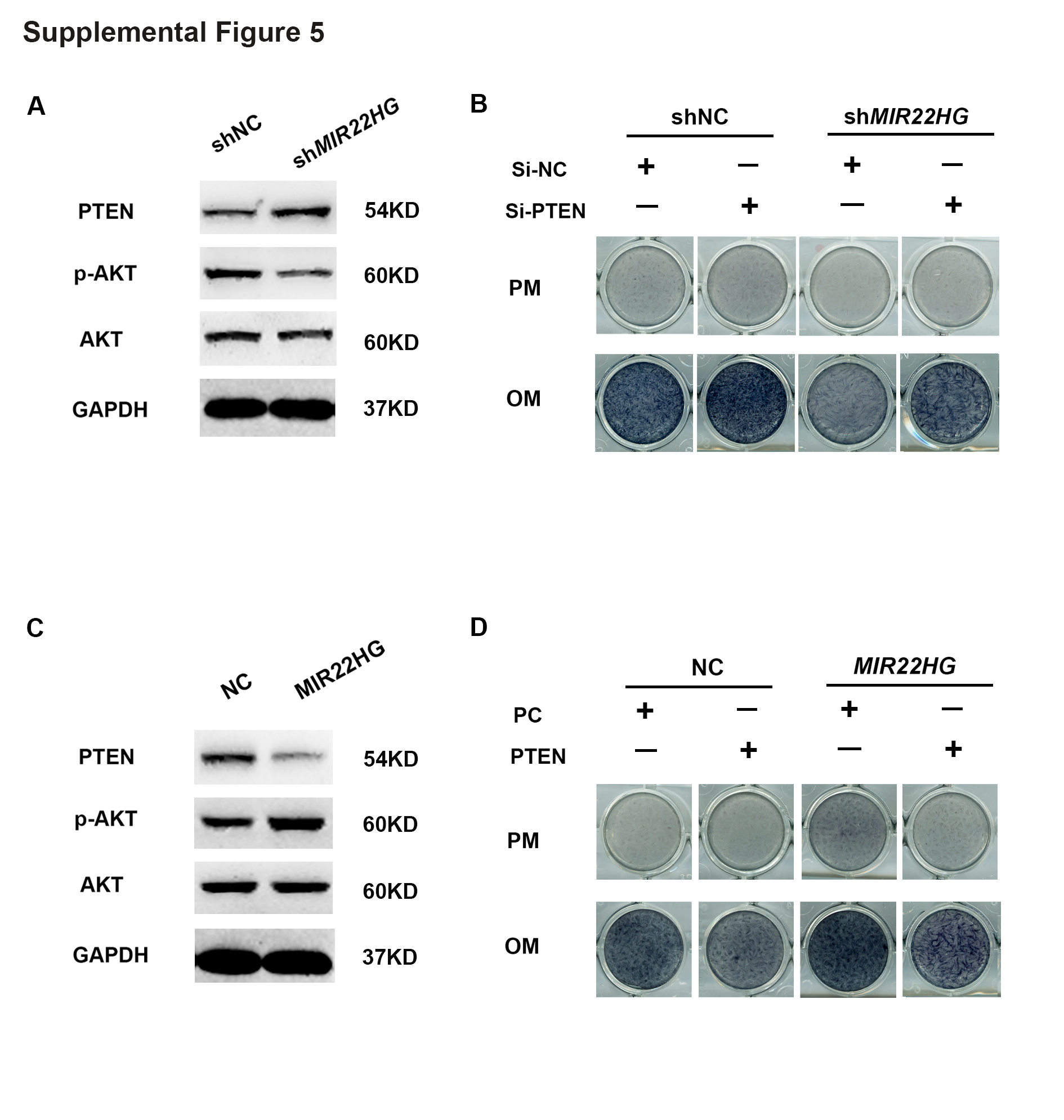

Supplement: Supplementary file 6 — Supplemental Figure 5 [file 41419_2020_2813_MOESM6_ESM.tif]

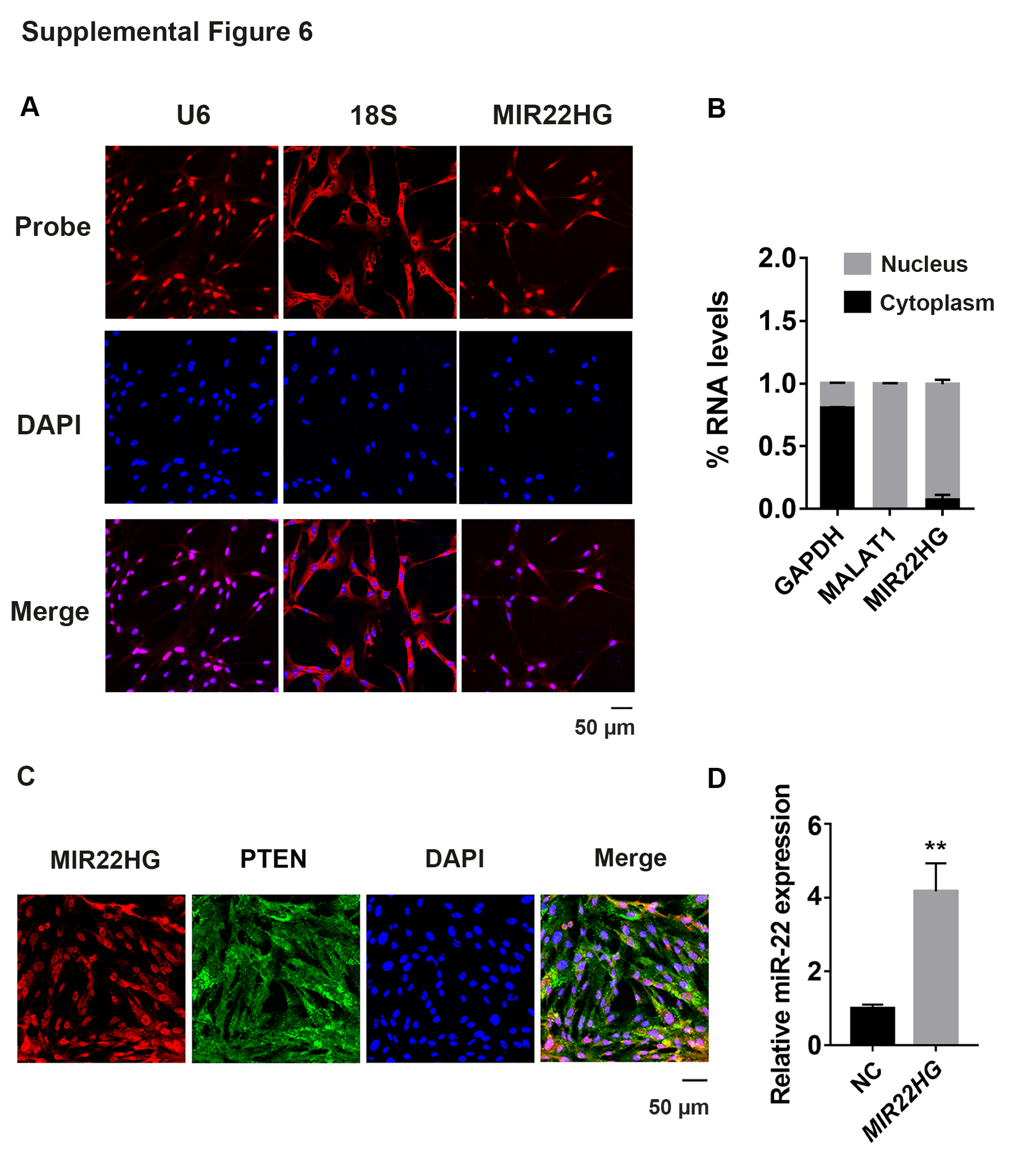

Supplement: Supplementary file 7 — Supplemental Figure 6 [file 41419_2020_2813_MOESM7_ESM.tif]

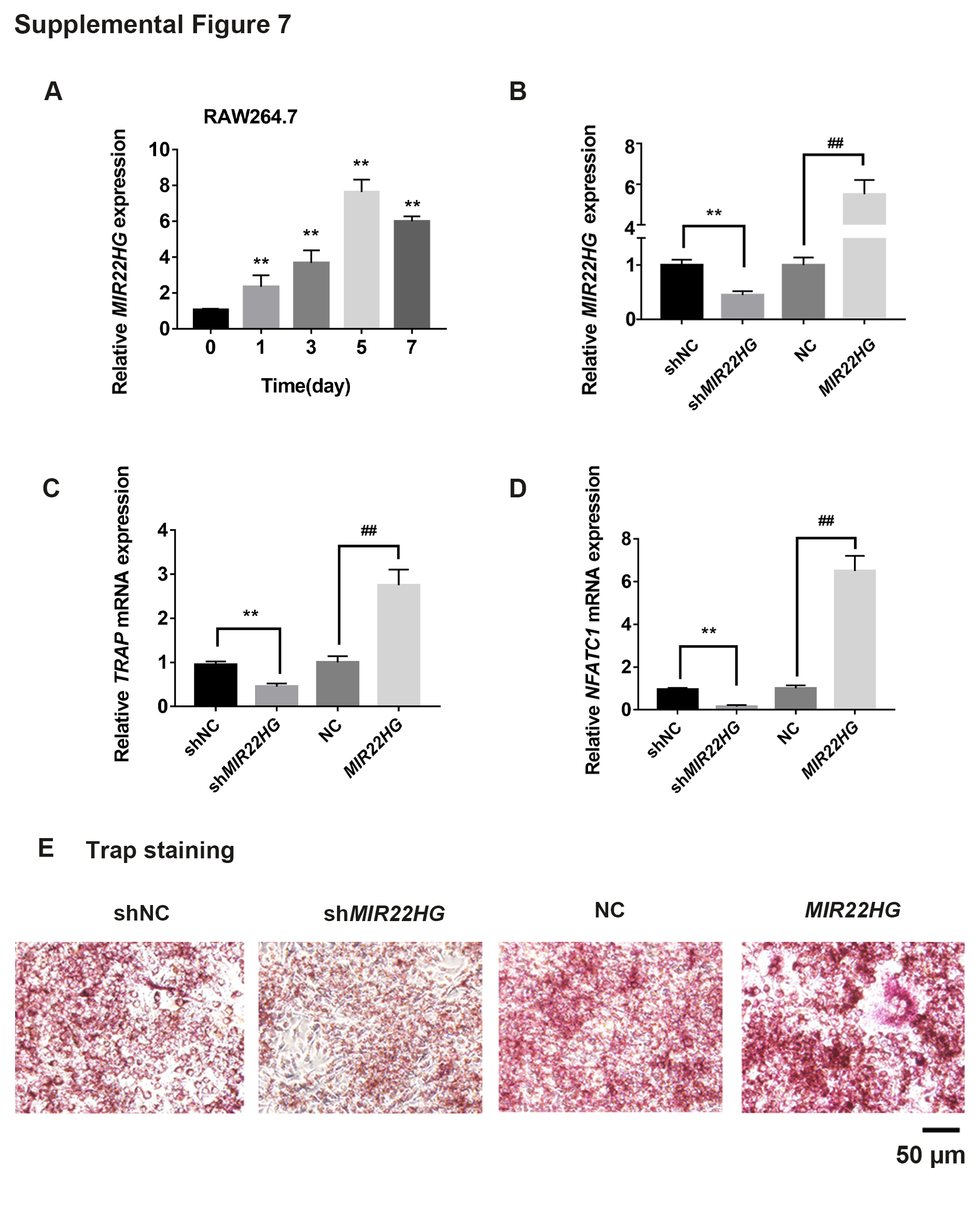

Supplement: Supplementary file 8 — Supplemental Figure 7 [file 41419_2020_2813_MOESM8_ESM.tif]
