# Supplementary material for: Long non-coding RNA MIR22HG promotes osteogenic differentiation of bone marrow mesenchymal stem cells via PTEN/ AKT pathway
Source: Cell Death Dis. 2020 Jul 30;11(7):601. doi: 10.1038/s41419-020-02813-2 (PMC7393093; doi:10.1038/s41419-020-02813-2)
Supplement: Supplementary file 9 — Supplemental table 1 [file 41419_2020_2813_MOESM9_ESM.doc]

Supplemental table 1. Sequences of RNA and DNA Oligonucleotides

| Name | Sense Strand/Sense Primer (5'-3') | Antisense Strand/Antisense Primer (5'-3') |
| --- | --- | --- |
| Primers for qRT-PCR | | |
| MIR22HG | AGCGGACGCAGTGATTTGCT | TGGCAGCTTTAGCTGGGTCA |
| ALP | ATGGGATGGGTGTCTCCACA | CCACGAAGGGGAACTTGTC |
| RUNX2 | CCGCCTCAGTGATTTAGGGC | GGGTCTGTAATCTGACTCTGTCC |
| OCN | CACTCCTCGCCCTATTGGC | CCCTCCTGCTTGGACACAAAG |
| GAPDH | GGTCACCAGGGCTGCTTTTA | GGATCTCGCTCCTGGAAGATG |
| MALAT1 | AAAGCAAGGTCTCCCCACAAG | GGTCTGTGCTAGATCAAAAGGCA |
| Mouse GAPDH | ACAGCAACTCCCACTCTTCCAC | AGTTGGGATAGGGCCTCTCTTG |
| Mouse RUNX2 | AAGTGTTCTGTGGTCTCTGAGTTGA | GCTGTATGGTGAGGCTGGTAGG |
| Mouse MIR22HG | CATCCGGAAGTGTGTCCCTC | CGTGCCTCTCTGACCAATGT |
| Mouse TRAP | CTGGAGTGCACGATGCCAGCGACA | TCCGTGCTCGGCGATGGACCAGA |
| Mouse NFATC1 | CCCGGAGTTCGACTTCGATT | TAACTGTAGTGTTCTGCGGC |
| siRNA | | |
| si-PTEN | CGAUAGCAUUUGCAGUAUAGA | UAUACUGCAAAUGCUAUCGAU |
| si-NC | UUCUCCGAACGUGUCACGUTT | ACGUGACACGUUCGGAGAATT |
| shRNA | | |
| sh*MIR22HG*-1 | GATGACAGTTGGGTGATAT |  |
| sh*MIR22HG*-2 | GTGGTTATCTTTGTATGGT |  |
| shNC | TTCTCCGAACGTGTCACGT |  |

Abbreviations: ALP, alkaline phosphatase; GAPDH, Glyceraldehyde 3-phosphate dehydrogenase; OCN, osteocalcin; RUNX2, runt-related transcription factor 2; PTEN, phosphatase and tension homolog; TRAP, tartrate resistant acid phosphatase; NFATC1, nuclear factor of activated T cells 1
